# FFU-Net: Feature Fusion U-Net for Lesion Segmentation of Diabetic Retinopathy

**DOI:** 10.1155/2021/6644071

**Published:** 2021-01-02

**Authors:** Yifei Xu, Zhuming Zhou, Xiao Li, Nuo Zhang, Meizi Zhang, Pingping Wei

**Affiliations:** ^1^School of Software, Xi'an Jiaotong University, 710054, Xi'an, Shaanxi, China; ^2^Huiyichen Inc. 1703, Block 1, No 1388, Jiulonghu Ave, 330038 Nanchang, Jiangxi, China; ^3^Baidu Inc. Baidu Building, 10 Shangdi 10th Street, Haidian District, 100080 Beijing, China; ^4^State Key Laboratory for Manufacturing Systems Engineering, Xi'an Jiaotong University, 710054, Xi'an, China

## Abstract

Diabetic retinopathy is one of the main causes of blindness in human eyes, and lesion segmentation is an important basic work for the diagnosis of diabetic retinopathy. Due to the small lesion areas scattered in fundus images, it is laborious to segment the lesion of diabetic retinopathy effectively with the existing U-Net model. In this paper, we proposed a new lesion segmentation model named FFU-Net (Feature Fusion U-Net) that enhances U-Net from the following points. Firstly, the pooling layer in the network is replaced with a convolutional layer to reduce spatial loss of the fundus image. Then, we integrate multiscale feature fusion (MSFF) block into the encoders which helps the network to learn multiscale features efficiently and enrich the information carried with skip connection and lower-resolution decoder by fusing contextual channel attention (CCA) models. Finally, in order to solve the problems of data imbalance and misclassification, we present a Balanced Focal Loss function. In the experiments on benchmark dataset IDRID, we make an ablation study to verify the effectiveness of each component and compare FFU-Net against several state-of-the-art models. In comparison with baseline U-Net, FFU-Net improves the segmentation performance by 11.97%, 10.68%, and 5.79% on metrics SEN, IOU, and DICE, respectively. The quantitative and qualitative results demonstrate the superiority of our FFU-Net in the task of lesion segmentation of diabetic retinopathy.

## 1. Introduction

Diabetic retinopathy is one of the main causes of blindness in human eyes, and regular fundus screening is an effective way to discover the location of disease [1–6]. At present, fundus screening is mainly diagnosed by analyzing fundus images manually, which requires ophthalmologists to have expert clinical experience. Therefore, the automatic screening and diagnosis of diabetic retinopathy have important practical significance. Moreover, the lesion segmentation of diabetic retinopathy is the prerequisite work for screening and diagnosing diabetic retinopathy, and it also lays a foundation for the subsequent grading of the severity of diabetic retinopathy. Generally, common diabetic retinopathy consists of microaneurysms (MA), hard exudates (EX), soft exudates (SE), and hemorrhage (HE).

In the past few decades, numerous researchers have devoted themselves to solving the segmentation of diabetic retinopathy. In early years, the researchers focused on traditional image processing methods, such as morphological operations and threshold segmentation [7–9]. Limited by the heavy dependence of the design level, the traditional methods of lesion segmentation are relatively infeasible in real-world application.

With the rapid development of deep learning technology, many researchers resort to deep learning methods to segment the lesion of diabetic retinopathy [3]. Although deep learning models can avoid handcrafted complex image features, it is difficult to segment tiny lesions composed of relatively macrostructures, such as microaneurysms and hemorrhage. As a classical medical semantic segmentation network, the symmetry-driven U-Net model [10] is weak in processing tiny lesions. In order to achieve more accurate results, we propose a deep neural model called FFU-Net with an encoder-decoder structure. In detail, the pooling layer of U-Net is substituted with a convolutional layer to reduce the spatial loss of the fundus image. For the purpose of extracting multiscale lesion features, the MSFF block is embedded in the encoder by considering splitting operations and residual modules into account. For the decoders, contextual channel attention modules is integrated with the concatenation of skip connection and lower-resolution decoder. To alleviate the imbalance problem between lesion area and normal area in a fundus image, an improved Focal Loss named Balanced Focal Loss is proposed to train our model. In comparison with the state of the art, the experimental results on the public IDRID demonstrate that our model surpasses other models on metrics SEN, IOU, and DICE.

Our contributions are summarized as follows: (1) We replace the pooling layer of U-Net with a convolutional layer for downsampling, which helps to preserve spatial loss of fundus images as much as possible. (2) In the encoders, we integrate MSFF block with U-Net to extract multiscale lesion features by taking splitting operation and residual module into account, which is beneficial to representing informative features. (3) In the decoders, we propose the CCA module to fuse the information between skip connection and lower-resolution decoder, which share attentions and enhance their representative ability efficiently. (4) We propose a new loss to address the imbalance data problem when training our model, which facilitates the discrimination ability of our model. (5) We conduct several evaluations of the comparative methods on the benchmark dataset to figure out the superiority of our model.

The rest of this paper is organized as follows. Materials and Methods displays the related work, methodology, and experiment settings. The experimental results and the discussion are presented in Results and Discussion. Finally, Conclusion and Future Work concludes our work and suggests possible topics for future research.

## 2. Materials and Methods

### 2.1. Related Work

In the early years, the medical researchers focused on the segmentation of diabetic retinopathy based on traditional digital image processing methods, such as morphological operations and threshold segmentation. For example, Fleming et al. [7] used morphological operations and Gaussian matched filters to extract candidate regions of microaneurysms and then collected various statistical features to eliminate false positive points in blood vessels, yielding accurate segmentation of microaneurysms. Antal and Hajdu [11] adopted an ensemble learning strategy to integrate a series of image preprocessing approaches to improve final segmentation of microaneurysms. Kavitha and Duraiswamy [8] extracted exudate features using a multilayer threshold method, but this model has requirements for the input image quality. In conclusion, the traditional methods of lesion segmentation are relatively inefficient with poor generalization.

Recently, the development of deep learning has been widely concerned in the field of medical treatment. Medical image segmentation [12] has also become a hot topic. Most existing models with excellent performance in medical image segmentation tasks are reconstructed based on FCN or U-Net. In FCN [13, 14], the last full connection layer was replaced with a convolution layer. Rather than a fixed input size required by the classical CNN model, it allowed input image with arbitrary size. Also, skip connections were employed to combine local information learned from shallow layers and complex information learned from deeper layers. In U-Net, a contracting path was used for capturing context and a symmetric expanding path is designed for precise localization. With reference to the upsampling strategy, FCN applied upsampling operation to the last feature map while U-Net transformed high-level features to low-level features by deconvolution operations. References [15, 16] advanced in U-Net by using max-pooling indices and multipath input, respectively. Van Grinsven et al. [17] sped up the training by dramatically selecting misclassified negative samples. Sambyal et al. [18] presented a modified U-Net architecture based on the residual network and employ periodic shuffling with subpixel convolution initialized to convolution nearest-neighbor resize.

### 2.2. Methodology

#### 2.2.1. Network Description

**Figure 1:**
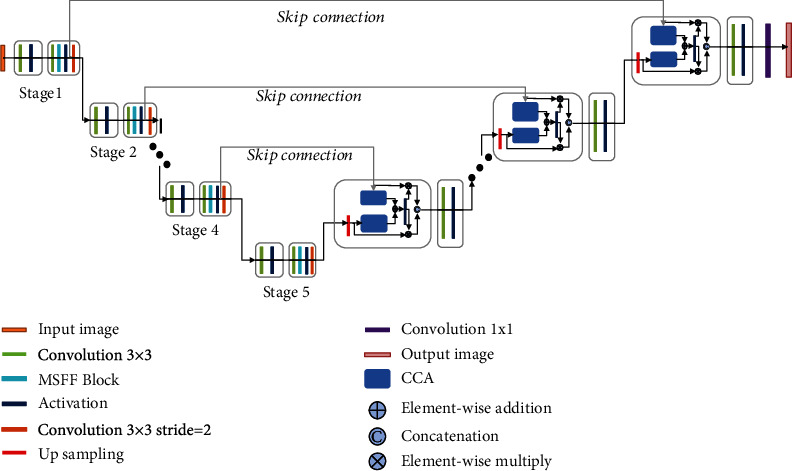
The overall architecture of the proposed FFU-Net model.

**Figure 2:**
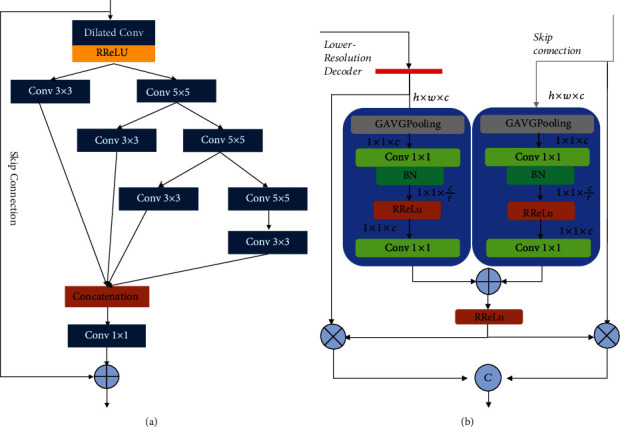
The improvements in the encoders and decoders of FFU-Net: (a) the structure of MSFF; (b) the CCA module in the decoders.

The overall pipeline of our proposed model is depicted in Figure 1. U-net was originally designed and developed for biomedical image segmentation. Its architecture is broadly regarded as an encoder network followed by a decoder network. For the encoder network, it is usually a pretrained classification network in which a downsampling pooling layer is appended at multiple different levels. For the decoder network, it includes upsampling and concatenation followed by regular convolution operations. The discriminative feature obtained by the encoder is projected onto pixel space to predict pixel-wise classification. As an extension of U-Net, our model makes the following three improvements adapted for lesion segmentation of diabetic retinopathy. (1) In the encoder stage, the maximum pooling layer of the original U-Net model for downsampling is substituted with a convolutional layer, in which the kernel size is 3 × 3 and stride = 2. The motivation behind this strategy could be explained as two points. (a) Compared with the pooling layer, downsampling with the convolution layer could keep structure information of diabetic retina images as much as possible. (b) It promotes the fusion of information between different channels, which is beneficial to the lesion segmentation task of diabetic retinopathy. Moreover, inspired by Inception block [19] and channel splitting idea [20], we design a new multiscale feature-fused block named MSFF to capture the features of the diabetic retinopathy image at different scales. As illustrated in Figure 2(a), the MSFF uses a series of multiscale residual splitting operations to extract different scale features. Firstly, as dilated convolution [21] could increase the receptive field under the condition that the resolution of the feature map is unchanged, we use a 3 × 3 dilated convolution followed by the RReLu layer to perceive more information. Then, we put forward a series of splitting steps to produce multiscale features efficiently. For each step, MSFF employs 3 × 3 and 5 × 5 convolution layers to split the preceding features into two parts. One part is retained, and the other part is fed into the next step. After three splitting steps, all the distilled features are concatenated together and then fed into a 1 × 1 convolution to reduce the channels and parameters. In our implementation, only 1/3 channels in each splitting step are kept. (2) In the decoder stage, the concatenation procedure between skip connection and lower-resolution decoder is improved with the contextual channel attention (CCA) module. We borrow the idea from SeNet [22] and depict the detail in Figure 2(b). Given lower-resolution decoder LD and skip connection S K with the size *h* × *w* × *c*, the proposed concatenation procedure with CCA can be described as

(1) $$\begin{array}{c} C_{\text{LD}}=\text{Conv}1\left(\text{RReLu}\left(\text{Conv}1\_\text{BN}\left(\text{GAPool}\left(\text{UP}\left(\text{LD}\right)\right)\right)\right)\right),\\ C_{\text{SK}}=\text{Conv}1\left(\text{RReLu}\left(\text{Conv}1\text{BN}\left(\text{GAPool}\left(\text{S K}\right)\right)\right)\right.,\\ \text{CCA}=\text{RReLu}\left(C_{\text{LD}}⊕C_{\text{SK}}\right),\\ F=\text{Concat}\left(\left(\text{CCA}⊗\text{LD}\right),\text{CCA}⊗\text{S K}\right), \end{array}$$

where UP and GAPool denote upsampling operation and global average pooling. Conv1_BN is the 1 × 1 convolution followed by batch normalization while Conv1 is the common 1 × 1 convolution. RReLu and Concat represent the RReLu activation function and concatenation operation along the channel dimension. After the GAPooling operation, 1 × 1 × (*c*/*r*) (*r* = 2) is employed to extract channel-wise statistics efficiently. As a contextual channel attention, CCA carries the channel-wise attentions from both LR and SK and then, respectively, multiply itself by LR and SK. Later, these two features are concatenated to replace the original concatenation procedure appearance in U-Net. In this way, LR and SK fully fuse the context information and share channel attention to provide more informative representation, which is conducive to the segmentation accuracy.

Besides, all the activation layers are replaced with nonlinear activation RReLu layers [23]. The reason why we prefer RReLu than other activation functions is that it could provide a random value from a uniform distribution to reduce overfitting during training. Herein, benefiting from the above-mentioned improvements, our FFU-Net achieves segmentation accuracy of the four lesions of diabetic retinopathy effectively.

#### 2.2.2. Loss Function

**Figure 3:**
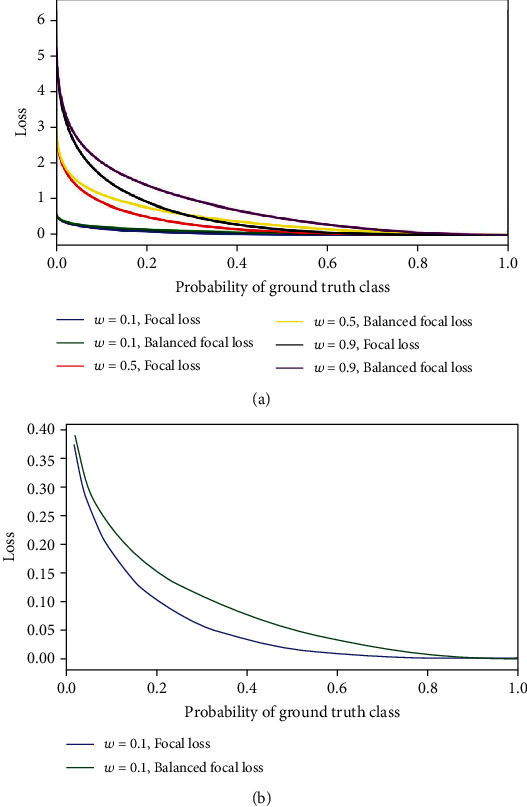
The comparative curves of Balanced Focal Loss and Focal Loss. (a) The results of Balanced Focal Loss and Focal Loss with different weights. (b) The zoom results of Balanced Focal Loss and Focal Loss when *w* = 1.

Apart from the network architecture, loss function also plays a key part in network design. In a diabetic retinopathy image, huge contrast could be found between the lesion and the normal from the perspective of appearance. Additionally, the size of the lesion area is always much smaller than the rest. Provided that we still insist on training our model to minimize the classification cross-entropy loss, the performance might not be like what it is supposed to be. This phenomenon can be ascribed to the imbalance problem occurring in the medical dataset. To address this issue, one can resort to data augmentation technology which duplicates samples to make the overall training set balanced. However, on account of the lack of diversity, the new dataset cannot provide clear improvement for our model. Alternatively, we turn to loss function according to the intrinsic distribution of data samples. Generally, the error penalties for the majority class and the minority class are different. Thus, we attempt to assign different weights to different classes and construct a Balanced Focal Loss for our model [24]. When training with this loss function, our model highlights the lesions of diabetic retinopathy. Different from original focal loss, in the task of medical segmentation in our application, the difference between easy and hard examples is more imperceptible. Mathematically, the loss function is formulated as follows:

(2) $$\begin{array}{c} L=\overset{n}{\underset{\text{i}=1}{\sum }}-w\left(\left|y-Q_{i}^{y}\right|\right)\left(\left(1-y\right)\log\left(1-Q_{i}\right)+y\log Q_{i}\right), \end{array}$$

where *n* represents the number of pixels in a diabetic retinopathy image and *i* denotes the *i*th sample. Here, ∣·∣ guarantees the nonnegativity. If the pixel is normal, its corresponding value is set to 0. If the pixel belongs to the lesion area, its corresponding value is set to 1. The parameter *w* represents the weight coefficient, which refers to the ratio between the pixels labeled as abnormal and the number of pixels in all samples. *Q*_*i*_ is the probability predicted by our proposed model; *γ* is the tunable focusing parameter which is always set to 2 in practice. As a comparison, we depict the values of Balanced Focal Loss and Focal Loss in Figure 3. As can be seen, when *Q*_*i*_ → 1 and *y* = 1, the loss for well-classifier examples is downweighted. For instance, when *y* = 1, an example with *Q*_*i*_ = 0.9 and *w* = 0.1 would be 5x lower (0.002) than cross-entropy (0.010). Although the case with Focal Loss shows 100x lower (0.0001), the gap between Balanced Focal Loss and Focal Loss is 0.0019. Besides, another example with *Q*_*i*_ = 0.1 and *w* = 0.1 generates 0.227, which is closer to the result of cross-entropy (0.230). By this means, this proposed Balanced Focal Loss increases the importance of correcting misclassified examples.

### 2.3. Data Preparation and Processing

#### 2.3.1. Data Preparation

**Figure 4:**
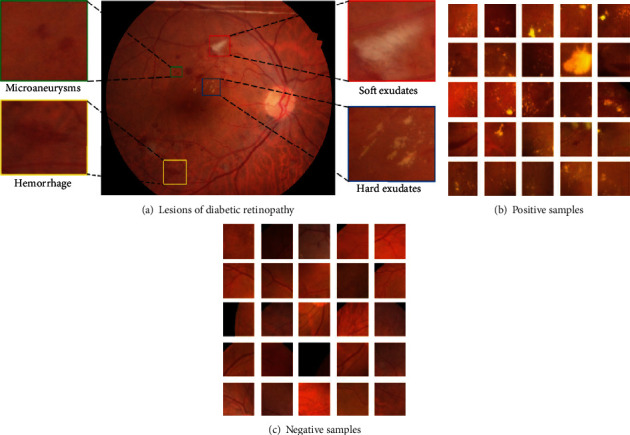
Color fundus samples of dataset IDRID: (a) the sample contains microaneurysms, hard exudates, soft exudates, and hemorrhage; (b) positive samples; (c) negative samples.

The dataset we adopted is the Indian Diabetic Retinopathy Image Dataset (IDRID) [25], which is derived from a patient's fundus image during a real clinical examination at an ophthalmology clinic in India. All images in the dataset were taken by a Kowa VX-10*α* color fundus camera with a 50-degree field of view close to the macular area. All images have a resolution of 4288 × 2848 in JPG format. In our experiment, we select 81 color fundus images from 516 images along with pixel-level annotations. As illustrated in Figure 4(a), four typical diabetic retinopathy abnormalities appear in this dataset. The IDRID is split into the training set and testing set according to different lesion labels. Empirically, the distribution results are displayed in Table 1.

#### 2.3.2. Fundus Image Preprocessing

A fundus image is taken with a color fundus camera. In most cases, influenced by uneven light intensity and camera lens contamination, the resultant fundus images are corrupted by uneven brightness, resulting in blurry and noisy areas. If the corrupted images are trained by the deep neural model directly, the noises will have adverse impact on the subsequent lesion segmentation of diabetic retinopathy.

**Figure 5:**
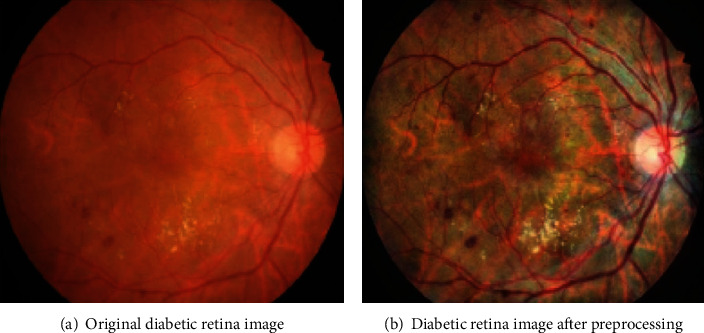
The comparison between the original diabetic retinopathy image and its corresponding preprocessed result.

To address the above problems, we take measures before feeding the fundus images into our network, such as image cropping, image denoising, image enhancement [26], image normalization, data augmentation, and image dicing. Here, we will illustrate the detail of preprocessing procedures. (1) Image cropping: the original samples are usually enclosed with a black border. To get the Region of Interest (ROI), OTSU and maximum connected components are used to obtain the optimal treatment threshold and remove outliers, respectively. (2). Image denoising: in the nature scenery, most photos are collected in Gaussian noise environment. To improve the robustness, Gaussian filter with 3 × 3 kernel is utilized to depress image noises. (3) Image enhancement: it can be observed that microaneurysms, hemorrhage, and blood vessel have indistinguishable appearance in color space. If one aims to enhance image quality towards the direction of color variance, it is in vain for recognizing the three objects. Therefore, CLAHE (Contrast Limited Adaptive Histogram Equalization) is applied to enhance images in contrast [27]. (4) Image normalization: considering that the color and brightness of fundus images are quite different, we need to confine some parameters in our network model to a reasonable range. Otherwise, the overlarge parameters will slow the convergence speed of our model. Thus, we use normalization operation to speed up and boost the performance of our model at the same time. Formally, the normalized image can be generated as follows:

(3) $$\begin{array}{c} x_{\mathrm{norm}}=\frac{x-u}{\theta }, \end{array}$$

where *x* and *x*_norm_ denote the original image and normalized image, respectively. *μ* and *θ* are the mean value and standard derivation of all the samples in dataset IDRID. (5) Data augmentation: in contrast with traditional RGB images, collecting medical images is arduous. However, the performance of a deep neural network relies heavy on the scale of training data. Hence, we resort to common-used data augmentation strategies: random horizon flips, rotation, random crop, shift, and rescaling. (6) Image dicing: as we can see, the resolution of the original image in dataset IDRID is 4288 × 2848, which hinders the deep model from running in low-capacity devices. Besides, the areas occupied by lesions are usually relatively small, and the locations of lesions are scattered. So, we resolve to improving the performance of our model via image dicing technology. In Figures 4(b) and 4(c), motivated by the sliding window method, the dataset is divided into positive samples (with lesions) and negative samples (without lesions). As depicted in Figure 5, the detailed characteristics of the lesion area are clear enough, which is conducive to the subsequent lesion segmentation.

After the above image preprocessing operations, as displayed in Figures 4(b) and 4(c), the original high-resolution fundus images are transformed into several subimages with 256 × 256 pixels using the sliding window strategy with stride = 64. Then, the subimages with a black background are eliminated, and the remaining are treated as the valid input.

#### 2.3.3. Fundus Image Postprocessing

After the above-mentioned image preprocessing, the whole image has been transformed into a group of subimages. For our trained model, the segmentation output has the same shape with the input subimage. Nevertheless, in real-world application, the pixels of the original image should be assigned with predicted labels in the final segmentation output. To achieve it, we attempt to merge these subimages to form the final segmentation result. The predicted label of a pixel is jointly determined by averaging the segmentation results of multiple subimages.

As mentioned in Fundus Image Preprocessing, the subimages are generated by the sliding window strategy. In this way, several subimages are overlapped inevitably. For the pixel inside the boundary, its final label will be assigned by averaging 16 subimage blocks. For the pixel on the boundary, it should be processed individually.

### 2.4. Experiments and Analysis

#### 2.4.1. Training Parameters

All the experiments are executed on hardware devices with Intel Xeon CPU, 128 GB memory, and NVIDIA Tesla P100 GPU. The software environment is Ubuntu 16.04 operating system and PyTorch 1.0 framework. The input size is 256 × 256, and the batch size is set to 64. Since no pretrained model is provided, He initialization is used to initialize our model [28]. The network is trained by optimizing loss *L* for 100 epochs. As we all know, a higher and fixed learning rate cannot guarantee to bring better convergence to the deep neural network. Consequently, we adjust the learning rate as the training procedure goes on. The initial learning rate is set to 2 × 10^3^. When the loss stops decreasing during training, the learning rate is reduced by a factor of 10. Also, the Adam optimizer with setting (*β*_1_ = 0.9, *β*_2_ = 0.999) is adopted. To be fair, all the comparative methods are implemented and line with the hyperparameters and parameters in their papers.

#### 2.4.2. Evaluation Metrics

Evaluation metrics play an important role in measuring the performance of comparative models. In order to analyze the experimental results quantitatively, we use several specific metrics to evaluate the performance in the task of segmenting diabetic retinopathy image, including Sensitivity (SEN), Intersection-over-Union (IOU), and Dice coefficient (DICE). To implement them, we first calculated true positive (TP), false positive (FP), true negative (TN), and false negative (FN). TP here refers to the intersection of the true lesion area and predicted lesion area, FP denotes the intersection of the true normal area and predicted lesion area, TN is the intersection of the true normal area and predicted normal area, and FN is defined as the intersection of the true lesion area and predicted normal area. Based on the above concepts, we introduce the following metrics:

(4) $$\begin{array}{c} \text{SEN }\left(\text{sensitivity}\right)=\frac{\text{TP}}{\text{TP}+\text{FN}},\\ \text{IOU}=\frac{\text{TP}}{\text{TP}+\text{FP}+\text{FN}},\\ \text{DICE}=\frac{2\text{TP}}{2\text{TP}+\text{FP}+\text{FN}}. \end{array}$$

Let us take a close look at the three metrics. Sensitivity can be treated as the misdiagnosis rate of a disease. In our work, it refers to the proportion of TP and true lesion area, which is a critical and foremost factor for patients and doctors. In real-world application, we try to decrease the misdiagnosis rate to the best of our ability. IOU is an evaluation metric used to measure the accuracy of a semantic segmentation model, and it specifies the amount of overlap between the predicted results and the groundtruth. DICE is a measure of how similar the prediction and groundtruth are, which not only is a measure of how many positives the models predict but also penalizes for the false positives of the models. Regarding the above commonly used metrics, the closer they are to 1, the better the segmentation performance.

## 3. Results and Discussion

In this section, we conduct our experiments to evaluate the performance of our segmentation methods. The experiments include three parts: the first part makes ablation study of our method. It demonstrates the different performance brought by the components appearing in our methods. The second part makes user study to evaluate our method against several state-of-the-art methods on dataset IDRID. The last part describes the parameters and costs of all the methods to verify their efficiency.

### 3.1. Ablation Study

To better evaluate our proposed method, we design an ablation study by replacing each component and keeping the rest unchanged. We place particular emphasis on differences brought by four improvements discussed in Network Description. Thus, we conduct the following experiments.

*Experiment 1*: the original U-Net model is trained and tested on our testing samples.

*Experiment 2*: based on the original U-Net, the cross-entropy loss is replaced with Balanced Focal Loss function (denoted as U-Net-FL for convenience).

*Experiment 3*: based on Experiment 2, the pooling layers in encoders are replaced with 3 × 3 convolution layers, and all the activation functions are set to RReLu (denoted as U-Net V1 for convenience).

*Experiment 4*: on the basis of Experiment 3, the MSFF block to extract multiscale features is integrated into encoders (denoted as U-Net V2 for convenience).

*Experiment 5*: on the basis of Experiment 4, the CCA module is deployed to fuse skip connection and lower-resolution decoder (denoted as FFU-Net for convenience).

All the above experiments are performed on a preprocessed dataset, and the quantitative results are illustrated in Tables 2 and 3. Obviously, FFU-Net consistently outperforms U-Net on all metrics in the task of segmenting lesions. This improvement is mainly attributed to MSFF, CCA, and Balanced Focal Loss. Using Balanced Focal Loss, U-Net-FL increase IOU by up to an average 0.031 points on all lesion types, which proves that Balanced Focal Loss function is capable of coping with data imbalance and misclassification in the segmentation task. After the pooling layers are replaced with 3 × 3 convolution layers and all the activations are set to RReLu, U-Net V1 achieve slightly better than U-Net-FL. The introduction of MSFF brings more improvement on metrics SEN, IOU, and DICE, which verifies the effectiveness of MSFF block to lesion segmentation for diabetic retinopathy. With the help of CCA, FFU-Net achieves the DICE value increased by 0.0291 points, and the IOU value increased by 0.0347 points. Note that in the analyses of CCA and MSFF, we find that they surpass U-Net V1 by a large margin on all metrics. This indicates that the components of CCA and MSFF play more critical roles in segmentation of medical images.

**Figure 6:**
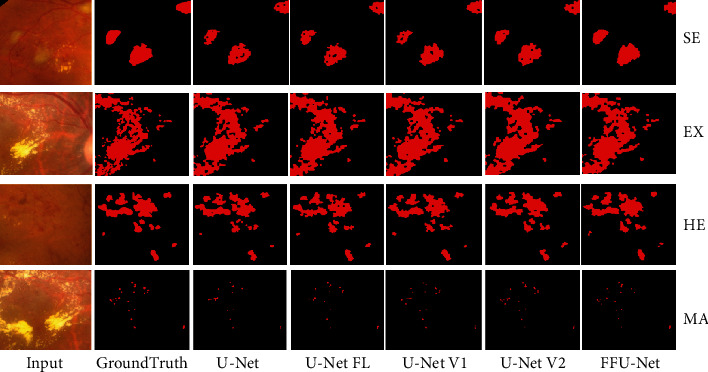
The visual segmentation results of U-Net, U-Net-FL, U-Net V1, U-Net V2, and FFU-Net. Zoom in to see the details.

In Figure 6, we visually present the segmentation results of different methods on dataset IDRID. It can be seen that U-Net and U-Net-FL cause too many defects with lower accuracy. Seen from the prediction results by U-Net V1, we observe that it can provide more clear boundaries than U-Net-FL. Since MSFF is utilized in the encoders, it appears that U-Net V2 produce clear and pleasing segmentation results. Nevertheless, we find that U-Net V2 fails to recognize the lesion with smaller size (MA). By incorporating the CCA module, FFU-Net aid in refining the details of lesions, leading to closer segmentation result to the groundtruth. Therefore, we can safely draw the conclusion that the improvements mentioned in FFU-Net are effective quantitatively and qualitatively.

### 3.2. User Study

**Figure 7:**
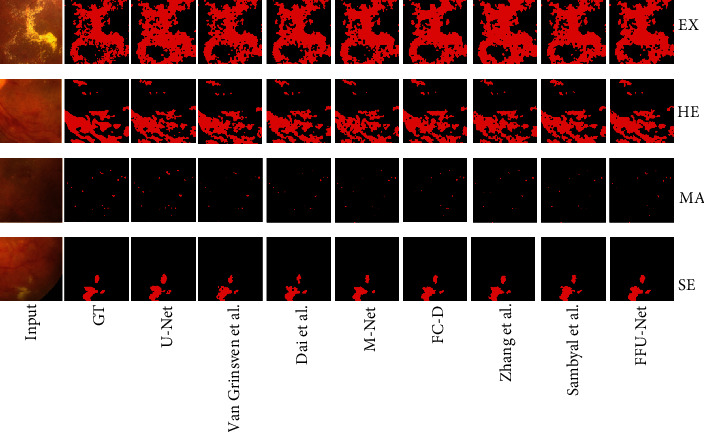
The visual comparative results for segmentation on dataset IDRID. GT: groundtruth; FC-D: FC-DenseNet. Zoom in to see the details.

To confirm the effectiveness and robustness of our proposed method, we conduct a user study against the state of the art on metrics SEN, IOU, and DICE. The comparative methods include the Dai et al. method [29], Zhang et al. method [30], Van Grinsven et al. method [17], M-Net [31], FC-DenseNet [32], Sambyal et al. method [18], and original U-Net. To further show our superiority, we, respectively, display the segmentation quantitative results on four lesion types in Tables 4 and 5. As can be seen, FFU-Net claims its superiority over the others on segmenting all the lesions. In comparison with the second best method (Sambyal et al.), FFU-Net achieves the DICE value increased by 2.0% and the IOU value increased by 3.5%. As reported in [29], the Dai et al. method is designed for timely detection and treatment of MA, which is consistent with our results in Table 5. However, it is unable to cope with the detection of other lesion types (EX and SE). Similarly, Zhang et al. aim to automatically detect exudates in color eye fundus images and perform better in segmenting EX and SE but work worse in segmenting MA and HE. Van Grinsven et al. solve the unbalanced problem by dynamically selecting misclassified negative samples and apply CNN to HE segmentation. The results reported in work [17] are verified in our experiment. Limited by the lack of generalization ability, Van Grinsven et al. are incapable of processing EX, SE, and MA perfectly. Although M-Net achieves state-of-the-art OD and OC segmentation results on the glaucoma dataset, it fails to transfer to our IDRID well. Besides, FC-DenseNet extends DenseNet to deal with the problem of semantic segmentation on natural images. When applying it to IDRID, it cannot show enough ability of presenting irregular microlesions. Sambyal et al. employ periodic shuffling with subpixel convolution initialized to convolution nearest neighbor resize. As we all know, the subpixel strategy is a common trick in the superresolution task. Whereas in Figure 7, we found more holes in the segmentation results, leading to unsatisfactory quantitative results on all metrics. Benefiting from the MSFF, CCA, and Balanced Focal Loss, our proposed FFU-Net achieves consistent improvement to all existing methods on all three performance metrics. Figure 7 shows some visual examples of four lesion types, where we observe that our method could generate closer results to the groundtruth without introducing additional artifacts. Apparently, we can see that Dai et al., Zhang et al., and Van Grinsven et al. suffer from inaccurate prediction for the boundaries of all lesion types. Also, the failure of M-Net and FC-DenseNet in transferring to all image samples is attributed to their poor generalization ability. Therefore, it can safely come to the conclusion that FFU-Net achieves comparable performance quantitatively and qualitatively.

### 3.3. The Overhead of Parameters and Computation

It is necessary to analyze the overhead of parameters and computation of our comparative methods. Notably, all comparisons are evaluated on the same machine. Evidently, as seen in Table 6, the Dai et al. method and Zhang et al. method are significantly lighter than other models, but this comes at the price of an apparent performance drop. With respect to the Van Grinsven et al. method, it solves the segmentation task through a CNN pixel-wise classifier. Whereas without taking spatial relationship into account, Van Grinsven et al. cannot achieve pleasing results. Since FC-DenseNet has more dense residual modules and more than 100 layers, it needs more time and more parameters in the testing procedure. As another modified U-Net, the Sambyal et al. method employs periodic shuffling with subpixel convolution based on U-Net, so it will take more time to implement in our application. By introducing splitting operation into FFU-Net, we observe that FFU-Net elapses less time while making noticeable improvement on segmentation performance. From the above discussions, it is observed that perhaps FFU-Net is the best choice when considering the influences between various factors.

## 4. Conclusion and Future Work

Based on the original U-Net network, we propose a new model named FFU-Net which is suitable for lesion segmentation of diabetic retinopathy. The FFU-Net network model mainly has the following contributions: The original pooling layer is replaced with a convolutional layer to reduce the spatial loss of the fundus image. MSFF block is incorporated to extract multiscale features and speed up feature fusion with splitting operation. By virtue of the CCA module, FFU-Net fuses the information between skip connection and lower-resolution decoder with shared attention weights. Considering the data imbalance problem in diabetic retinopathy, we present a Balanced Focal Loss function. Finally, in order to verify the effectiveness of our proposed model, ablation study and user study are carried out on the public benchmark IDRID. The final experimental results demonstrate the effectiveness and advancement of our proposed FFU-Net in terms of almost all metrics.

In the future, we will investigate a more general and comprehensive segmentation method for diabetic retinopathy and put emphasis on the following points: (1) Few-shot learning: though we solve the overfitting problem caused by insufficient data by data slicing, the burden of collecting large-scale supervised data for real-world application is still challenging. Thus, we resort to few-shot learning to achieve better segmentation. (2) Contaminated labels: different from the benchmark that is refined and maintained by professionals, the practical images of diabetic retinopathy are vulnerable to be contaminated and damaged. Thus, we should learn how to segment the lesion images only with incomplete and contaminated labels. (3) Grading the severity of diabetic retinopathy: as a foundation work, we plan to expand our work to grade the severity of diabetic retinopathy and apply our achievements to real-world application.

## Figures and Tables

**Table 1:** The distribution of IDRID.

Lesion type	Training set	Testing set
Microaneurysms	54	27
Hard exudates	54	27
Soft exudates	26	14
Hemorrhage	53	27

**Table 2:** Ablation study of the proposed model against U-Net, U-Net V1, U-Net V2, and U-Net-FL on EX and SE.

		EX			SE	
Methods	SEN	IOU	DICE	SEN	IOU	DICE
FFU-Net	0.8755	0.8414	0.9138	0.7933	0.7876	0.8812
U-Net V2	0.8440	0.8159	0.8986	0.7547	0.7535	0.8594
U-Net V1	0.8033	0.7867	0.8769	0.6934	0.7028	0.8191
U-Net-FL	0.7929	0.7763	0.8704	0.6801	0.6893	0.8099
U-Net	0.7819	0.7602	0.8638	0.6713	0.6707	0.8029

**Table 3:** Ablation study of the proposed model against U-Net, U-Net V1, U-Net V2, and U-Net-FL on MA and HE.

		MA			HE	
Methods	SEN	IOU	DICE	SEN	IOU	DICE
FFU-Net	0.5933	0.5610	0.7188	0.7342	0.7365	0.8450
U-Net V2	0.5508	0.5267	0.6669	0.6936	0.6917	0.8177
U-Net V1	0.5172	0.4891	0.6334	0.6598	0.6562	0.7897
U-Net-FL	0.4968	0.4626	0.6255	0.6447	0.6425	0.7797
U-Net	0.4810	0.4490	0.6197	0.6366	0.6333	0.7755

**Table 4:** Comparative segmentation results of the proposed model against the state of the art on EX and SE.

		EX			SE	
Methods	SEN	IOU	Dice	SEN	IOU	DICE
Dai et al.	0.8074	0.7843	0.8791	0.7006	0.7071	0.8284
Zhang et al.	0.8418	0.8137	0.8973	0.7523	0.7505	0.8575
Van Grinsven et al.	0.8031	0.7749	0.8732	0.6988	0.692	0.818
M-Net	0.8327	0.8083	0.894	0.7297	0.7156	0.8343
FC-DenseNet	0.8414	0.8099	0.8949	0.7554	0.7623	0.8651
Sambyal et al.	0.8421	0.8183	0.9001	0.7563	0.763	0.8656
FFU-Net	0.8755	0.8414	0.9138	0.7933	0.7876	0.8812
U-Net	0.7819	0.7602	0.8638	0.6713	0.6707	0.8029

**Table 5:** Comparative segmentation results of the proposed model against the state of the art on MA and HE.

		MA			HE	
Methods	SEN	IOU	DICE	SEN	IOU	DICE
Dai et al.	0.5498	0.5237	0.6874	0.6895	0.6990	0.8228
Zhang et al.	0.4897	0.4723	0.6416	0.6418	0.6407	0.7810
Van Grinsven et al.	0.4832	0.4667	0.6364	0.6844	0.6761	0.8068
M-Net	0.5366	0.5097	0.6753	0.6872	0.6796	0.8093
FC-DenseNet	0.5521	0.5276	0.6908	0.6976	0.6960	0.8208
Sambyal et al.	0.5537	0.5438	0.7045	0.6998	0.7038	0.8261
FFU-Net	0.5933	0.5610	0.7188	0.7342	0.7365	0.8450
U-Net	0.4810	0.4490	0.6197	0.6366	0.6333	0.7755

**Table 6:** The overhead of parameters and computation of different comparative models.

Models	Running time	Parameters
Dai et al. method	616 ms	—
Zhang et al. method	688 ms	—
Van Grinsven et al. method	2598 ms	0.98 M
M-Net	3745 ms	1.67 M
FC-DenseNet	4361 ms	1.73 M
Sambyal et al. method	1535 ms	1.33 M
FFU-Net	695 ms	0.97 M
U-Net	780 ms	1.93 M

## Data Availability

The source code data used to support the findings of this study are available from the corresponding author upon request.
